# Validation of the Charlotte Large Artery Occlusion Endovascular Therapy Outcome Score in a Modern Cohort of Thrombectomy Patients

**DOI:** 10.3390/neurolint17080130

**Published:** 2025-08-21

**Authors:** Rahul R. Karamchandani, Liang Wang, Hongmei Yang, Dale Strong, Jeremy B. Rhoten, Jonathan D. Clemente, Gary Defilipp, Elizabeth A. Adelman, William R. Stetler, Andrew W. Asimos

**Affiliations:** 1 Department of Neurology, Neurosciences Institute, Wake Forest University School of Medicine, Advocate Health, Charlotte, NC 28203, USA; 2 Clinical Quality Analytics, Advocate Health, Charlotte, NC 28203, USA; liang.wang1@advocatehealth.org (L.W.); hongmei.yang@advocatehealth.org (H.Y.); edwin.strong@advocatehealth.org (D.S.); 3 Neurosciences Institute, Advocate Health, Charlotte, NC 28203, USA; jeremy.rhoten@advocatehealth.org (J.B.R.); elizabeth.adelman@advocatehealth.org (E.A.A.); 4 Charlotte Radiology, Neurosciences Institute, Wake Forest University School of Medicine, Advocate Health, Charlotte, NC 28203, USA; jonathan.clemente@charlotteradiology.com (J.D.C.); gary.defilipp@charlotteradiology.com (G.D.); 5 Carolina Neurosurgery & Spine Associates, Department of Neurosurgery, Neurosciences Institute, Wake Forest University School of Medicine, Advocate Health, Charlotte, NC 28203, USA; will.stetler@cnsa.com; 6 Department of Emergency Medicine, Neurosciences Institute, Wake Forest University School of Medicine, Advocate Health, Charlotte, NC 28203, USA; andrew.asimos@advocatehealth.org

**Keywords:** stroke, endovascular thrombectomy, outcome prediction

## Abstract

Background/Objectives: The Charlotte Large artery occlusion Endovascular therapy Outcome Score (CLEOS) predicts neurological outcomes after endovascular thrombectomy (EVT). Given recent expanded indications for EVT, we evaluated CLEOS in a modern cohort of thrombectomy patients. Methods: We retrospectively analyzed consecutive, anterior circulation EVT patients from January to December 2024 at multiple centers. The primary outcome was a 90-day modified Rankin Scale (mRS) score of 4–6. We compared primary outcome rates between the original CLEOS derivation cohort and the validation cohort. The area under the curve (AUC) was calculated for CLEOS and compared to other prognostic scales. Results: In the 347 included patients, the mean age was 67.6 (14.9) years, the median National Institutes of Health Stroke Scale (NIHSS) was 15 (10–20), and 137 (42.2%) had a 90-day mRS score of 4–6. A similar proportion of patients in the validation cohort and the derivation cohort achieved the primary outcome (39% each, *p* = 0.957). The AUC for CLEOS (0.7416, 95% confidence interval [CI] 0.688–0.795) was superior to that of the Pittsburgh Response to Endovascular therapy (AUC 0.681, 95% CI 0.624–0.738, *p* < 0.01) and Stroke Prognostication using Age and NIHSS (AUC 0.5982, 95% CI 0.556–0.640, *p* < 0.01), while a trend was observed compared to Houston Intra-Arterial Therapy-2 (AUC 0.6999, 95% CI 0.644–0.756, *p* = 0.0657) and Totaled Health Risk in Vascular Events (AUC 0.7046, 95% CI 0.560–0.759, *p* = 0.07). CLEOS ≥ 700 predicted the primary outcome in 16/19 (84.2%) patients. Conclusions: CLEOS performed well in our modern cohort of thrombectomy patients. Prognostic scales such as CLEOS may be useful in guiding conversations and setting expectations with family members pre- and post-thrombectomy.

## 1. Introduction

The Charlotte Large artery occlusion Endovascular therapy Outcome Score (CLEOS), first described in 2022 based on historical data from 2016 to 2020, predicts neurological outcomes after anterior circulation endovascular thrombectomy (EVT) [1]. It incorporates four, pre-thrombectomy variables–age; presenting National Institutes of Health Stroke Scale (NIHSS) score; initial glucose; and cerebral blood volume (CBV) index, derived from automated computed tomography perfusion (CTP) software–to quantify the likelihood of a poor neurological outcome (modified Rankin Scale [mRS] score 4–6) at 90 days [1]. The model has been evaluated in various cohorts, including patients presenting with a basilar thrombosis [2].

In this analysis, we evaluate CLEOS in a multicenter cohort of recent EVT patients. Since the derivation of CLEOS, notable changes in acute stroke care include the widespread use of tenecteplase rather than alteplase as the intravenous thrombolytic of choice [3], demonstration of the efficacy of thrombectomy versus medical management for large core stroke [4], and failure to show a benefit of thrombectomy in patients with medium-vessel occlusions [5,6]. In addition, with expanding EVT indications and a general increase in patient volumes, stroke interventionalists at our centers, as at other centers around the country, have gained invaluable experience. Given this changing climate, serial validation studies remain essential, as they serve to either strengthen confidence or identify gaps in a given prediction model.

## 2. Materials and Methods

We retrospectively analyzed all consecutive, anterior circulation EVT patients presenting to our health system from 1 January 2024 to 31 December 2024. The primary outcome was a 90-day mRS score of 4–6. The study was approved by the Wake Forest University School of Medicine Institutional Review Board (IRB00082295) and deemed exempt from requiring subject consent given its retrospective nature.

Data was abstracted from the code stroke registry for the health system, which includes two thrombectomy centers: a Joint commission-certified Comprehensive Stroke Center and a Thrombectomy Capable Center. Demographics and details of a patient’s medical history, including hypertension, hyperlipidemia, diabetes mellitus, coronary artery disease, and smoking, were included. Vessel occlusions were categorized by the most proximal site of thrombosis. Computed tomography (CT) Alberta Stroke Program Early CT Score (ASPECTS) were calculated by a neuroradiologist and CTP parameters were recorded from automated software (RAPID AI, San Mateo, CA, USA). Endovascular thrombectomy was performed according to our health system guidelines (Figure S1). An intracranial hemorrhage was defined as symptomatic if it resulted in a 4-point worsening in the NIHSS within 36 h. Excellent revascularization was defined as modified treatment in cerebral ischemia (mTICI) 2c-3. Modified Rankin Scale scores were generally captured via a standardized telephone questionnaire, as the code stroke registry for the health system is prospectively maintained. In rare instances when phone contact could not be made, a board-certified vascular neurologist (R.R.K.) or a comprehensive stroke center coordinator (J.B.R) performed a chart review within the 90 ± 15-day window to obtain the mRS.

Baseline characteristics were compared between the validation and original CLEOS derivation cohorts. Characteristics were displayed as counts (percentage), mean (standard deviation [SD]), or median (interquartile range [IQR]). The chi-square test, Mann–Whitney U test, and Fisher’s exact test were used to compare characteristics, as appropriate, with a *p*-value < 0.05 considered to be statistically significant.

CLEOS, defined as (5 × age [years]) + (10 × NIHSS) + glucose (milligrams/deciliter)–(150 × CBV index), was calculated for each patient in the validation cohort. Rates of poor 90-day functional outcome (mRS 4–6) were calculated by CLEOS group (<400, 400–499, 500–599, 600–699, ≥700) and compared to the original derivation cohort. The positive predictive value for a CLEOS of ≥700 to predict a 90-day mRS score of 4–6 was calculated, given the cutoff of 700 previously described as a potential marker of futile recanalization [1]. Receiver operator characteristics (ROC) curves were constructed and the area under the curve (AUC) was calculated for CLEOS and compared to previously described prognostic models: Totaled Health Risks in Vascular Events (THRIVE) [7], Houston Intra-Arterial Therapy-2 (HIAT-2) [8], Pittsburgh Response to Endovascular therapy (PRE) [9], and Stroke Prognostication using Age and NIHSS (SPAN-100) [10], to predict 90-day mRS 4–6 (Table S1).

## 3. Results

A total of 371 patients were treated with EVT during the study period, 24 of whom had basilar thrombosis, leaving 347 anterior circulation thrombectomy patients in the validation cohort. The mean age was 67.6 (14.9) years, 180 (51.9%) were male, and the median NIHSS was 15 (10–20) (Table 1). Mean time to skin puncture was 476 (445) minutes. Excellent revascularization was achieved in 175 (50.4%) patients and 137 (42.2%) had a 90-day mRS score of 4–6 (Table 1). Differences between the derivation and validation cohorts were present for rates of comorbid coronary artery disease, presenting NIHSS, last known well (LKW) to skin puncture time, and treatment with intravenous thrombolysis (Table 1).

**Figure 1:**
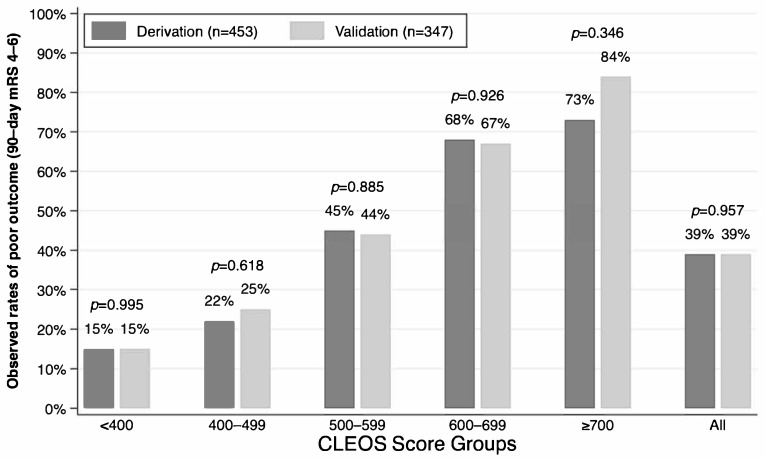
Rates of poor 90-day outcome stratified by CLEOS groups. CLEOS, Charlote Large artery occlusion Endovascular therapy Outcome Score; n, number; mRS, modified Rankin Scale.

Figure 1 shows the rates of poor outcomes (90-day mRS 4–6) stratified by CLEOS groups. A similar proportion of patients in the validation cohort had poor outcomes compared with the derivation cohort overall and in each score group (<400, 400–499, 500–599, 600–699, ≥700). Of the 19 patients with CLEOS ≥ 700, 16 (84.2%) had 90-day mRS scores of 4–6.

**Figure 2:**
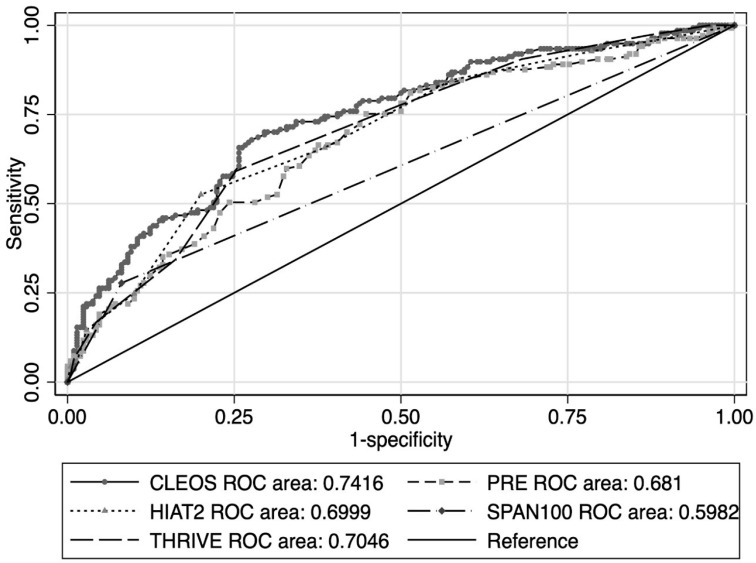
Receiver operator characteristics curves for outcome prediction models. CLEOS, Charlote Large artery occlusion Endovascular therapy Outcome Score; ROC, receiver operator characteristics; PRE, Pittsburgh Response to Endovascular therapy, HAT2, Houston Intra-Arterial Therapy-2; SPAN100, Stroke Prognostication using Age and National Institutes of Health Stroke Scale; THRIVE, Totaled Health Risks in Vascular Events.

The ROC curves are displayed in Figure 2. The AUC for CLEOS (0.7416, 95% confidence interval [CI] 0.688–0.795) was superior to that of PRE (AUC 0.681, 95% CI 0.624–0.738, *p* < 0.01) and SPAN-100 (AUC 0.5982, 95% CI 0.556–0.640, *p* < 0.01), while it was numerically higher than, though not statistically different to, that of HIAT-2 (AUC 0.6999, 95% CI 0.644–0.756, *p* = 0.0657) and THRIVE (AUC 0.7046, 95% CI 0.560–0.759, *p* = 0.07).

## 4. Discussion

In a 1-year cohort of thrombectomy patients from modern clinical practice, the outcome prediction model CLEOS performed comparably well to its performance in the original derivation cohort, which was based on patients treated from the years 2016 to 2020. Specifically, observed rates of poor neurological outcome (90-day mRS 4–6) were similar for the entire validation cohort and in each CLEOS group, while the AUC in the validation cohort (0.7416) was nearly identical to that reported in the derivation cohort (0.7482). Scores of 700 or greater were associated with a high likelihood of non-ambulatory status, total dependency, or death (90-day mRS 4–6).

The ever-evolving nature of acute stroke care mandates continuous evaluation and refinement of outcome prediction models. Despite notable changes in the field, including widespread use of intravenous tenecteplase rather than alteplase, an increased volume of thrombectomies performed in large core infarction patients, and a reduction in medium-vessel occlusion thrombectomy, CLEOS predicted poor outcomes in our modern thrombectomy cohort comparably well to the previous report. Of note, all subjects treated with intravenous thrombolysis in the validation cohort received tenecteplase, while those in the derivation cohort were treated with alteplase. Despite similar CT ASPECTS and CTP core sizes in the cohorts, comparable numbers of medium and distal branch middle cerebral artery occlusions treated, and only minimal differences in presenting stroke severity (NIHSS 15 in the validation cohort compared to 16 in the derivation cohort), patients in the validation cohort had higher rates of coronary artery disease, significantly longer times to skin puncture, and were less frequently treated with intravenous thrombolysis. The performance of CLEOS in our study speaks to its durability in a generally less favorable cohort of patients.

While numerous large-vessel occlusion prediction models and scores have been reported in the literature [11], CLEOS is unique in its construction. It incorporates only four variables, allowing for relative ease of calculation. Three of these four are part of a standard stroke evaluation (age, NIHSS, glucose), and the fourth (CBV index) does not require any manual calculation or subjective judgment, as do other markers, such as angiographic collateral scores. In addition, the elements of CLEOS are all available before a thrombectomy, allowing for risk stratification prior to EVT, compared to other models that incorporate procedural or post-acute findings. Lastly, as reported in the original manuscript [1], it offers stratification based on an assumption of achieving excellent revascularization, allowing for a best-case prognosis to be obtained.

Previous studies have also explored the performance of CLEOS as a predictive tool [1,2]. Given its strong prognostic ability in anterior circulation thrombectomy, CLEOS was also evaluated in patients with basilar artery thrombosis [2]. Compared with a CTP-based model that quantifies severe hypoperfusion in the vertebrobasilar distribution (the critical area perfusion score [CAPS]), CLEOS performed better than CAPS at predicting 90-day mRS scores of 4–6 [2]. In the subset of patients with good reperfusion after thrombectomy, CLEOS demonstrated a greater sensitivity for predicting 90-day mRS 4–6 (71% versus 21%, *p* = 0.003). Twenty-two of the twenty-three (95.6%) subjects with CLEOS > 503 were neurologically dependent (mRS 3–6) at 90 days [2].

As described previously [1,12], prognostic models are a valuable tool for risk stratification, but should not be the only factor considered in clinical decision-making. While additional validation studies strengthen confidence in a model, the absence of any perfect tool allows for unexpected outcomes to occur, even when unlikely. In addition, even in cases when an “expected” poor outcome occurs after being predicted with high probability, this may be acceptable to the patient (i.e., mRS 4). Of note, the median 90-day mRS score in a pooled analysis of large core thrombectomy patients was 4 [4], which is generally considered to be a poor outcome, but may be viewed positively in the setting of a large core infarction. Patients treated with thrombectomy in large core trials reported better overall quality of life in TENSION [13] and LASTE [14] and improved mobility, depression, and social domains in SELECT2 [15], despite median 90-day mRS scores of 4 in each treatment group. Even when a devastating outcome seems likely (mRS 5–6), this does not preclude offering thrombectomy as a treatment option, considering the poor natural history of patients treated with medical management in this scenario.

Our study is limited by several factors. Although we included patients treated at two thrombectomy centers, each operated under the same patient selection guidelines, so evaluating CLEOS outside of our health system would strengthen the findings. Patients considered poor candidates for thrombectomy based on various other factors may have been managed medically, creating a selection bias, as only thrombectomy patients were included. Subjects treated in a single year were studied, limiting the sample size, and specifically limiting patients in individual CLEOS groups. However, our intention was to evaluate patients from modern daily practice, which was achieved, though core sizes and the distribution of large- and medium-vessel occlusions were similar between the cohorts. Lastly, our findings are subject to the biases associated with any retrospective analysis, including the potential for unmeasured confounders to impact outcomes, though CLEOS performed similarly well in the validation cohort despite the presence of unmeasured confounders.

## 5. Conclusions

In conclusion, the prognostic tool CLEOS performed similarly well in our modern cohort of thrombectomy patients compared to its prognostic ability in the originally reported derivation cohort, despite differences in the presenting characteristics of the patients. Elevated scores are associated with worse 90-day functional outcomes, and scores of 700 or greater have a high probability of resulting in non-ambulatory status, total dependency, or death. Nonetheless, CLEOS, and other prognostic tools, have limitations, and may predict an outcome that is generally regarded as poor though is acceptable to the patient or their family. As such, CLEOS may be useful as one factor in medical decision-making and can help guide conversations and set expectations for family members pre- and post-thrombectomy.

## Figures and Tables

**Table 1:** Patient characteristics in derivation and validation cohorts ^a^.

	Derivation, n = 453	Validation, n = 347	*p*-Value ^b^
Age, years, mean ± SD	66.3 (15.4)	67.6 (14.9)	0.23
Sex, male, n (%)	230 (50.8%)	180 (51.9%)	0.76
Hypertension, n (%)	326 (72.0%)	245 (70.6%)	0.67
Hyperlipidemia, n (%)	199 (43.9%)	154 (44.4%)	0.90
Diabetes mellitus, n (%)	117 (25.9%)	85 (24.5%)	0.65
Coronary artery disease, n (%)	43 (9.5%)	65 (18.7%)	<0.05
Atrial Fibrillation, n (%)	138 (30.5%)	93 (26.8%)	0.26
Smoking, n (%)	170 (37.5%)	146 (42.1%)	0.19
Site of Occlusion, n (%)			0.37
Internal Carotid Artery	110 (24.3%)	83 (23.9%)	
Middle Cerebral Artery—M1	253 (55.8%)	178 (51.3%)	
Middle Cerebral Artery—M2	87 (19.2%)	84 (24.2%)	
Middle Cerebral Artery—M3	3 (0.7%)	2 (0.6%)	
Initial NIHSS, median (IQR)	16.0 (11.0–21.0)	15.0 (10.0–20.0)	<0.05
Glucose (mg/dL), mean ± SD	136.7 (59.3)	133.8 (50.3)	0.48
CT ASPECTS, median (IQR)	10.0 (9.0–10.0)	10.0 (8.0–10.0)	0.80
CBF < 30% (mL), median (IQR)	8.0 (0.0–30.0)	9.0 (0.0–35.0)	0.40
Tmax > 6 s (mL), median (IQR)	125.0 (83.0–176.0)	117.5 (73.0–181.0)	0.65
HIR, median (IQR)	0.5 (0.2–0.6)	0.5 (0.2–0.6)	0.49
CBV index, median (IQR)	0.8 (0.6–0.8)	0.7 (0.6–0.8)	0.41
LKW to skin puncture (min), mean ± SD	389.9 (331.4)	476.0 (445.0)	<0.05
Intravenous thrombolysis, n (%)	185 (40.8%)	109 (31.4%)	<0.05
Symptomatic ICH, n (%)	10 (2.2%)	5 (1.4%)	0.60
Post-treatment mTICI score 2c-3, n (%)	243 (53.6%)	175 (50.4%)	0.37
Poor 90-day mRS (4–6), n (%)	178 (39.3%)	137 (42.2%)	0.42

^a^ The values in Table 1 are based on observed data; missing values are not imputed. ^b^ *t*-test, Chi-square test, rank-sum test, and Fisher’s exact test were used to calculate the *p*-value. n, number; SD, standard deviation; NIHSS, National Institutes of Health Stroke Scale; IQR, interquartile range; mg, milligrams; dL, deciliter; CT, computed tomography; ASPECTS, Alberta Stroke Program Early Computed Tomography Score; CBF, cerebral blood flow; ml, milliliters; Tmax > 6 s, time-to-maximum greater than 6 s; HIR, hypoperfusion intensity ratio; CBV, cerebral blood volume; LKW, last known well; min, minutes; ICH, intracranial hemorrhage; mTICI, modified treatment in cerebral ischemia; mRS, modified Rankin Scale.

## Data Availability

The original contributions presented in this study are included in the article. Further inquiries can be directed to the corresponding author.

## Supplementary Materials

The following supporting information can be downloaded at: https://www.mdpi.com/article/10.3390/neurolint17080130/s1, Figure S1: Health System Endovascular Thrombectomy Guidelines; Table S1: Stroke Outcome Prediction Scales. Reference [16] are cited in Supplementary Materials.

## Abbreviations

The following abbreviations are used in this manuscript:

CLEOS	Charlotte Large artery occlusion Endovascular therapy Outcome Score
EVT	endovascular thrombectomy
NIHSS	National Institutes of Health Stroke Scale
CBV	cerebral blood volume
CTP	computed tomography perfusion
mRS	modified Rankin Scale
CT	computed tomography
ASPECTS	Alberta Stroke Program Early Computed Tomography Score
SD	standard deviation
IQR	interquartile range
ROC	receiver operator characteristics
AUC	area under the curve
THRIVE	Totaled Health Risks in Vascular Events
HIAT-2	Houston Intra-Arterial Therapy-2
PRE	Pittsburgh Response to Endovascular therapy
SPAN-100	Stroke Prognostication using Age and NIHSS
LKW	last known well
ml	milliliters
OR	odds ratio
