# Supplementary material for: Validation of the Charlotte Large Artery Occlusion Endovascular Therapy Outcome Score in a Modern Cohort of Thrombectomy Patients
Source: Neurol Int. 2025 Aug 21;17(8):130. doi: 10.3390/neurolint17080130 (PMC12388991; doi:10.3390/neurolint17080130)
Supplement: Supplementary file 1 [file neurolint-17-00130-s001.zip › neurolint-3786057-supplementary.pdf]

Figure S1. Health System Endovascular Thrombectomy Guidelines

EVT Guidelines

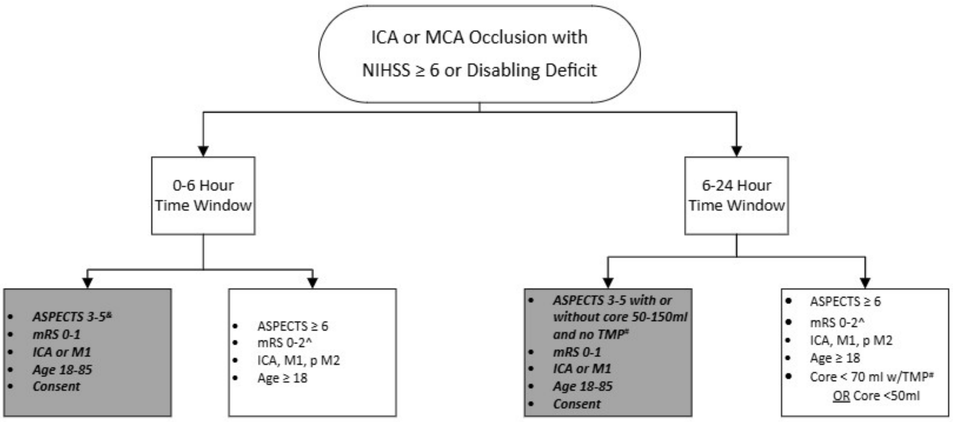

Gray shaded boxes indicate large core criteria

Exclusions: current hemodialysis, metastatic cancer, ventricular assist device, life expectancy < 1 year from underlying medical condition

&If CTP performed, core < 150 ml
#TMP = target mismatch profile: mismatch volume ≥ 15ml <u>and</u> mismatch ratio ≥1.8
^If mRS 3-4 or unknown, and all other radiographic criteria met, consider for EVT

The above serves only as a guideline. Decision making for acute stroke treatment is complex and best made by the treating team on an individual patient basis.

EVT = endovascular thrombectomy; ICA = internal carotid artery; MCA = middle cerebral artery; NIHSS = National Institute of Health Stroke Scale; ASPECTS = Alberta stroke program early CT score; mRS = modified Rankin Scale; M1 = M1 segment of MCA; p M2 = proximal M2 segment of MCA; TMP = target mismatch profile; CTP = computed tomography perfusion
