# Supplementary material for: Validation of the Charlotte Large Artery Occlusion Endovascular Therapy Outcome Score in a Modern Cohort of Thrombectomy Patients
Source: Neurol Int. 2025 Aug 21;17(8):130. doi: 10.3390/neurolint17080130 (PMC12388991; doi:10.3390/neurolint17080130)
Supplement: Supplementary file 1 [file neurolint-17-00130-s001.zip › Supplemental Table S1.pdf]

Table S1. Stroke Outcome Prediction Scales*

Scale Name	Calculation	Interpretation
Charlotte Large artery occlusion Endovascular therapy Outcome Score (CLEOS)[1]	(5 x age [years]) + (10 x NIHSS) + Glucose (mg/dL) – (150 x CBV index)	Higher scores associated with poor outcome (90-day mRS 4-6). Scores ≥ 700 associated with no statistical benefit of excellent endovascular reperfusion (mTICI 2c-3) in original derivation manuscript.
Totaled Health Risks in Vascular Events (THRIVE)[2]	Age 60-79 years, 1 point; age ≥80 years, 2 points; NIHSS 11-20, 2 points; NIHSS ≥21, 4 points; hypertension, diabetes mellitus, atrial fibrillation, 1 point each	Points (range 0-9)<br>Score 6-9 associated with poor 90-day outcomes.
Houston Intra-Arterial Therapy (HIAT)-2[3]	Age ≤59 years, 0 points; age 60-79 years, 2 points; age ≥80 years, 4 points; glucose < 150 mg/dL, 0 points, glucose ≥150 mg/dL, 1 point; NIHSS ≤10, 0 points; NIHSS 11-20, 1 point; NIHSS ≥21, 2 points; ASPECTS 8-10, 0 points; ASPECTS ≤7, 3 points	Points (range 0-10)<br>Score ≥5 associated with poor 90-day outcomes.
Pittsburgh Response to Endovascular therapy (PRE)[4]	Age (years) + (2 x NIHSS) – (10 x ASPECTS)	-25 to +49, likely to benefit from successful EVT; ≥ 50 not likely to benefit from successful EVT.
Stroke Prognostication using Age and NIHSS (SPAN-100)[5]	Age (years) + NIHSS	Score ≥100 associated with poor outcomes.

*Reproduced with permission from Elsevier: Karamchandani RR, Satyanarayana S, Yang H, et al. The Charlotte Large Artery Occlusion Endovascular Therapy Outcome Score Predicts Poor Outcomes 1 Year After Endovascular Thrombectomy. *World Neurosurg* 2023; 173: e415-e421.

NIHSS, National Institutes of Health Stroke Scale; mg, milligrams; dL, deciliters; CBV, cerebral blood volume; mRS, modified Rankin Scale; mTICI, modified thrombolysis in cerebral infarction; ASPECTS, Alberta Stroke Program Early Computed Tomography Score; EVT, endovascular thrombectomy

[1] Karamchandani RR, Prasad T, Strong D, et al. A tool to improve stroke outcome prediction: The charlotte large artery occlusion endovascular therapy outcome score. *J Stroke Cerebrovasc Dis* 2022; 31: 106393.

[2] Flint AC, Cullen SP, Faigeles BS, et al. Predicting Long-Term Outcome after Endovascular Stroke Treatment: The Totaled Health Risks in Vascular Events Score. *American Journal of Neuroradiology* 2010; 31: 1192–1196.

[3] Sarraj A, Albright K, Barreto AD, et al. Optimizing prediction scores for poor outcome after intra-arterial therapy in anterior circulation acute ischemic stroke. *Stroke* 2013; 44: 3324–3330.

[4] Rangaraju S, Aghaebrahim A, Streib C, et al. Pittsburgh Response to Endovascular therapy (PRE) score: optimizing patient selection for endovascular therapy for large vessel occlusion strokes. *J Neurointerv Surg* 2015; 7: 783–788.

[5] Saposnik G, Guzik AK, Reeves M, et al. Stroke Prognostication using Age and NIH Stroke Scale: SPAN-100. *Neurology* 2013; 80: 21–28.
